# Lm-LLO immunotherapies targeting multiple antigens and their impact on different mechanisms in the tumor microenvironment

**DOI:** 10.1186/2051-1426-2-S3-P63

**Published:** 2014-11-06

**Authors:** Anu Wallecha, Poonam Molli

**Affiliations:** 1 Advaxis, Inc, Princeton, NJ, USA

## 

Overexpression of tumor associated antigens (TAA) such as carbonic anhydrase 9 (CA9), HER2/neu and high molecular weight melanoma associated antigen (HMW-MAA) is associated with aggressive high-grade tumors leading to disease progression and reduced survival. CA9 is a cell surface enzyme that catalyzes the reversible hydration of carbon dioxide to bicarbonate and is overexpressed in response to tumor hypoxia in many common tumor types. CA9 plays a critical role in hypoxia-associated tumor acidosis, which plays an important role in tumor progression and chemoresistance in various types of cancer. Current HER2/neu-directed therapies confer limited clinical benefits and most patients experience progressive disease indicating that additional therapeutic strategies targeting HER2/neu could have potential. HMW-MAA is reported to be a TAA as well as an angiogenesis associated protein, as it is expressed at high levels by activated pericytes and pericytes in tumor angiogenic vasculature that are associated with neovascularization *in vivo*. We hypothesized that an *Lm*-LLO immunotherapy, using attenuated *Listeria moncytogene*s (*Lm*)-LLO as the vector capable of delivering multiple antigens would likely have a synergistic effect on decreasing tumor growth by targeting independent mechanisms that support tumor growth. We created two bivalent *Lm*-LLO immunotherapies expressing two antigens such as HER2/HMW-MAA or HER2/CA9. These bivalent *Lm*-LLO immunotherapies efficiently secreted two antigens, grew intracellularly and escaped the phagolysosome, supporting that recombinant bacteria retained their ability to deliver antigen successfully in an antigen presenting cell. Preliminary antitumor therapeutic studies in the treatment of mice bearing established tumors expressing HER2 demonstrate that both of these bivalent *Lm*-LLO immunotherapies show an improvement in the reduction of tumor growth when compared to monovalent *Lm*-LLO immunotherapies. We will present data on the therapeutic efficacy of two bivalent *Lm*-LLO immunotherapies and provide evidence on the mechanisms likely responsible for the observed anti-tumor effects. Currently *Lm*-LLO immunotherapies are being evaluated in Phase II clinical trials for HPV-associated malignancies such as cervical, head and neck, and anal cancers.

